# Combination of ketamine and xylazine exacerbates cardiac dysfunction in severely scalded rats during the shock stage

**DOI:** 10.3892/etm.2013.1213

**Published:** 2013-07-10

**Authors:** YONGQIANG FENG, JIAKE CHAI, WANLI CHU, LI MA, PEIPEI ZHANG, HONGJIE DUAN

**Affiliations:** Department of Burns and Plastic Surgery, The First Affiliated Hospital of Chinese PLA General Hospital, Beijing 100048, P.R. China

**Keywords:** anesthetics, cardiac dysfunction, burns, mitochondrial apoptosis, rat

## Abstract

Cardiac inhibition due to burn injury and anesthetics have been documented previously. However, little is known about their combined effects on cardiac function. The aim of the present study was to observe the effects of a ketamine/xylazine (K/X) combination on the cardiac function of rats with severe scalds and compare them with those of avertin. Adult rats were randomly distributed into four groups: the KXB group (scalds anesthetized with K/X, n=10), the KXC group (sham scalds anesthetized with K/X, n=10), the AVB group (scalds anesthetized with avertin, n=10) and the AVC group (sham scalds anesthetized with avertin, n=10). Ketamine and xylazine were administered at 25 and 6 mg/kg, respectively, and avertin at 200 mg/kg before full-thickness scalds or sham scalds of 30% total body surface area (TBSA) were produced. Echocardiographic parameters were assessed following injury. The heart rate (HR) in the KXB group was fatally low during the study period. Fractional shortening (FS%) and ejection fraction (EF) in the KXB group were extremely low initially and remained low. The left ventricular end-diastolic volume (LVEDV) and left ventricular end-systolic volume (LVESV) were reduced in the burned rats. Serum levels of cardiac troponin I (cTnI) were significantly higher in the KXB group than in the AVB group (1.66±0.28 vs. 1.16±0.34 ng/ml, P<0.01). The highest lung wet/dry weight ratio was observed in the KXB group. However, no evident heart tissue pathological changes were observed in these groups. The apoptotic index of myocardial cells and caspase 3 expression level were highest in the KXB group (P<0.01). In conclusion, K/X exacerbated cardiac inhibition in severely scalded rats during the shock stage by a mechanism which may involve mitochondrial apoptosis.

## Introduction

Cardiac dysfunction, an important characteristic of the acute phase response following severe burns (1), may trigger multiple organ failures during the shock stage and lead to poor outcomes. The pathophysiology of cardiac dysfunction following burn injury is not yet understood. Animals are required in order to study the mechanisms of action and develop new treatment strategies. Anesthesia is also required for the development of animal models of thermal injury and to perform other protocols.

Anesthetics may induce pathophysiological changes in hemodynamics, inflammation, oxidative stress and adhesion, and may even affect the progression of experimentally mimicked disease (2). Therefore, suitable anesthetics are important in animal experiments and allow adequate differentiation in application. Various anesthetics have been used in previous studies, including intraperitoneal injection of ketamine and xylazine (K/X), avertin (AV; also named tribromoethanol), chloral hydrate, barbiturates and thiobutabarbital, and inhaled volatile anesthetics, such as isoflurane and halothane (3–5). Among these, the K/X combination is one of the most widely used anesthetic approaches in animal experiments (6,7). In this combination, each component is suggested to compensate for the limitations of the other and to provide the most favorable anesthetic effect. Several authors, however, have reported that K/X may produce evident cardiac depression, including bradycardia and hypotension, in mice (8,9). AV is a frequently used, short-acting, alcohol-based anesthetic, which was demonstrated to induce only modest effects on M-mode estimates of basal cardiac function and have no effect on cardiac output (10). AV was used as the control anesthetic in the current study because of its relative cardiac advantages.

Echocardiography, a well-established non-invasive procedure, has been widely used for the detection of cardiac structure or cardiovascular effects (11–13). To the best of our knowledge, there have been no studies concerning its use in rats with burns to assess cardiac function. Although the effects of K/X and AV on cardiac function have been compared in detail by transthoracic echocardiography and closed-chest cardiac catheterization in normal adult male Swiss Webster mice (14), there have been no studies regarding the application of echocardiography in the assessment of cardiovascular changes in severely burned rats.

Troponin I is present in heart muscle tissue and is a highly specific and sensitive cardiac biomarker. Raised serum levels indicate cardiac injury and are associated with worse outcomes in numerous disease states (15). In trauma, raised cardiac troponin I (cTnI) levels have been identified at relatively late time points following injury and were observed to be associated with increased risks of adverse cardiac events and mortality (16). In the current study, the serum cTnI level was determined as a direct marker of myocardial injury.

Increased apoptosis of cardiomyocytes and the possible effects of this have been documented previously (17). In addition, the mitochondrial-mediated pathway of apoptosis may play a significant role in *in vivo* cardiac ischemia/reperfusion (18).

As previously stated, anesthetics and thermal injury may have synergistic impact on cardiac function. The present study used echocardiography and the determination of cTnI and apoptosis levels to assess the *in vivo* cardiac effects of K/X injections in severely scalded rats and to explore the preliminary mechanism.

## Materials and methods

### Animals

Forty male Wistar rats (Animal Research Laboratories of the First Affiliated Hospital of Chinese PLA General Hospital, Beijing, China) weighing between 200 and 220 g were kept under controlled standard housing conditions with free access to standard laboratory food and water for a 7-day adaptation period before being randomly assigned to different groups. The groups were as follows: KXB (scalds anesthetized with K/X, n=10), KXC (sham scalds anesthetized with K/X, n=10), AVB (scalds anesthetized with AV, n=10) and AVC (sham scalds anesthetized with AV, n=10).

### Ethical approval

All experiments in this study were conducted in accordance with the National Regulations for the Administration of Affairs Concerning Experimental Animals (approved by the State Council on October 31, 1988 and promulgated by Decree No. 2 of the State Science and Technology Commission on November 14, 1988) and the Beijing Regulations for the Administration of Affairs Concerning Experimental Animals (approved by the Science and Technology Committee of Beijing on October 17, 1996), and were approved by the Animal Protocol Review Board of Agents and Anesthesia Protocol of the ETM-2111 112383_Feng_08-03-2013-(E)-Jen/ce.

### Anesthetics and anesthesia protocol

The following agents were purchased: xylazine hydrochloride injection (1.5 ml:0.03 g, Sumianxin II; Shengda Animal Medicine Co. Ltd., Dunhua, Jilin, China), ketamine hydrochloride injection (2 ml:1 g; Fujian Gutian Pharmaceutical Co. Ltd., Ningde, China), AV (25 g, Sigma-Aldrich, St. Louis, MO, USA), t-amyl alcohol (100 ml, Sigma-Aldrich) and buprenorphine HCl (20 mg; Amresco LLC, Solon, OH, USA). AV was prepared as described previously (19).

Food was withheld for 12 h prior to the experimental procedures. On the day of the experiment, each rat was weighed and clinically examined for behavior, respiration and cardiovascular parameters. The experiments were conducted between 9:00 a.m. and 12:00 a.m. Two different regimens using intraperitoneal injections of a K/X mixture (25/6 mg/kg) or AV (200 mg/kg s.c.) were used to induce surgical-depth anesthesia in the rats. A standardized pain protocol was used to uniformly assess discomfort following injury and supplemental analgesics were administered accordingly. Buprenorphine (0.025–0.05 mg/kg s.c.) was administered when the animals showed signs of discomfort.

The rats then immediately underwent scalds or sham scalds, followed by echocardiography 10, 20 and 30 min after the scalds. The animals were sacrificed by decapitation using a standard small-animal guillotine device 24 h after scalding. Blood, lung and heart tissues were harvested.

### Scald procedure and resuscitation

Each rat was shaved in preparation for the experiments 1 day in advance. The animals were secured in a constructed wooden template device. The dorsal and lateral skin surfaces were exposed through an oval aperture in the template, and the animals were immersed in 94°C water for 12 sec on the back and upper sides as described previously (20).

The use of the template limited the burned area, avoided injury to the abdominal organs and produced full-thickness dermal scalds affecting 30% of the total body surface area (TBSA). The rats with sham scalds were handled identically to the scalded rats with the exception that they were immersed in room temperature water and thus served as controls. Following immersion in water, all rats were immediately dried, administered fluid (Ringer’s lactate solution, 4 ml/kg by the Parkland formula) (21) during the post-burn period and placed in individual cages awaiting echocardiography. The animals were maintained under anesthesia for the duration of the echocardiographic examination.

### Echocardiography

Hair was removed from the chests of the rats using a razor. The rats were then fixed in a left lateral position and placed on a heating pad to maintain the body temperature at 37–38°C. Acoustic coupling gel warmed to room temperature was applied to the chest prior to examination. A commercially available ultrasound system was used for the echocardiographic examinations (Sonos 7500; Philips, Andover, MA, USA). The depth was set to 2 cm and zoomed to 1.2 cm. Wall thickness and left ventricular (LV) dimensions were obtained from a short-axis view at the level of the papillary muscles at a frame rate of 260 Hz. The ultrasound system was used to measure the cardiac cycle (time), LV end-diastolic chamber diameter (LVEDd), LV end-systolic chamber diameter (LVESd), fractional shortening (FS%) and ejection fraction (EF). The Teichholz formula (22) was used to calculate the LV chamber volume [LV end-diastolic volume (LVEDV)]: LVEDV=[7.0/(2.4 + LVEDd)] × LVEDd^3^, where the heart rate (HR)=1/time × 60.

The gains were adjusted to eliminate background noise and enable clear tissue signals to be obtained; 5–10 cycles were recorded. The measurements and analyses were repeated by different individuals.

### Lung wet/dry weight and heart histopathological examination

The lung tissue samples from the study groups and from non-survivors were analyzed. The lung tissue was analyzed to determine the wet/dry weight ratio (W/D). Following the termination of the experiments, the lungs were removed from the perfusion chamber and weighed. The lungs were then heated in an oven at 80°C for 48 h and reweighed.

For histopathological analysis, a small sample of the heart was resected, fixed with 10% formaldehyde solution for 48 h, embedded in paraffin, cut into 4-μm pieces using a microtome, and stained with hematoxylin and eosin.

### cTnI analysis

The peritoneal cavity was opened to expose the coeliac artery, and blood samples were collected and centrifuged (2,000 × g, 4°C, 10 min) to separate the serum. The serum was collected and stored at −80°C until analysis. Commercially available ELISA kits (E02T0491; Shanghai BlueGene Biotech Co., Ltd., Shanghai, China) were used to determine serum cTnI levels according to the manufacturer’s instructions. A multidetection microplate reader (Synergy^TM^ 2; Biotek, Winooski, VT, USA) was used to detect cTnI activity at 460 nm after reacting the serum sample on a cTnI antibody-coated plate.

### Apoptosis (TUNEL) assay

The rat heart was excised and dissected longitudinally to expose the endocardium. Tissues were quickly fixed in 4% paraformaldehyde. The 5-μm thick paraffin-embeded sections were prepared for the TUNEL (Keygen Biotech, Nanjing, China) assay according to the manufacturer’s instructions. The sections were examined with a microscope at ×400 magnification. A total of 10 fields per section were examined by an investigator blinded to the experimental procedure. The brown-stained nuclei were regarded as cells undergoing apoptosis (positive cells). The apoptosis index (AI) was calculated using the following formula: AI (%)=N_positive cell_/N_total cell_×100%.

### Western blot analysis

The muscle tissues were frozen and stored in liquid N_2_ and total protein extracts were prepared using RIPA lysis buffer plus protease inhibitors, as described previously (20). Each protein lysate (40 mg) was separated by 15% sodium dodecyl sulfate-polyacrylamide gel electrophoresis (SDS-PAGE) and transferred to a PVDF membrane using a semi-dry system. Nonspecific sites were blocked with 5% nonfat dry milk in TBS containing 0.1% Tween-20 (TBS-T). The blots were incubated overnight with the appropriate dilution of the primary antibodies. Anti-bcl, bax, caspase 3 and cytochrome c (Cell Signaling Technology, Inc., Danvers, MA, USA) antibodies were used as primary antibodies at a dilution of 1:1,000. The membranes were repeatedly washed with TBS-T prior to incubation with HRP-conjugated anti-rabbit or anti-mouse IgG (Bioss, Beijing, China) antibody at a dilution of (1:2,000). The ECL blot detection kit (Thermo Scientific, Middletown, VA, USA) was used according to the manufacturer’s instructions to visualize reactive products.

### Statistical analysis

Statistical comparisons were performed only for exploratory data analysis. All values are presented as the mean ± SEM. The survival rates were evaluated using the Kaplan-Meier method. Analysis of variance was performed to assess an overall difference among the groups. The least significant difference method was used for pairwise multiple comparisons. SPSS for Windows (version 16.0; SPSS, Inc., Chicago, IL, USA) was used to perform all analyses. P<0.05 was considered to indicate a statistically significant result.

## Results

### Mortality

**Figure 1:**
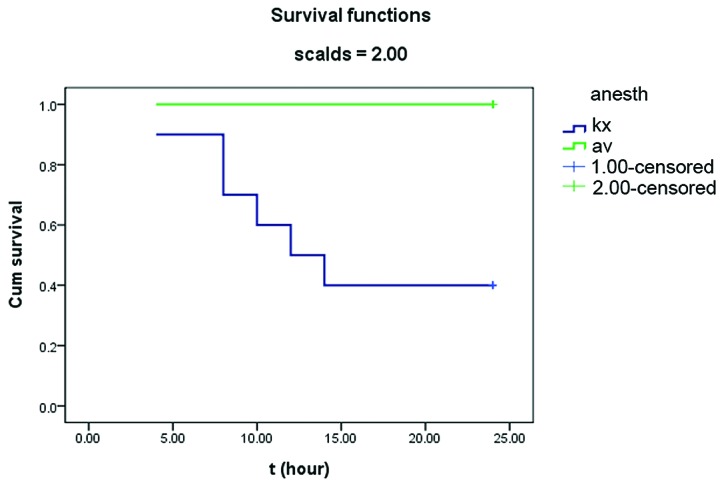
Survival analysis of scalded rats. The rats were anesthetized with avertin (AV) or a ketamine-xylazine combination (KX). The blue line represents the survival state of scalded rats treated with combination of KX within 24 h post-injury. The green line represents the survival state of scalded rats treated with AV within 24 h post-injury. There is a significant difference in the 24-h survival rate between the KX- and AV-treated rats (P<0.01). Censored, terminal time (24 h post-injury) of observation in this study; Cum survival, cumulative survival 24 h post injury; 1.00-censored, the terminal observation point of rats treated with KX; 2.00-censored, terminal observation point of rats treated with AV.

Survival rates were observed at 24 h after scalding. All burned and control rats anesthetized with AV had uniform recoveries, but those anesthetized with K/X were dispirited within 6–8 h after injury. Six rats (6/10, 60%) succumbed within 6–24 h after injury in the KXB group and one rat succumbed 4 h after sham injury in the KXC group. No rats anesthetized with AV died. As a result, the survival of the KXB group was significantly different (P=0.004) from that of the AVB group, as shown by the survival curves (Fig. 1).

### Comparison between the two anesthetic regimes in normal rats

**Figure 2:**
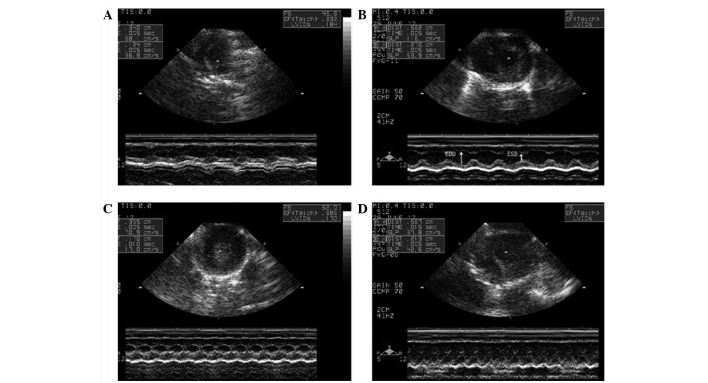
Representative M-mode images showing cardiac function and dimension in rats. The images represent the (A) KXB, (B) KXC, (C) AVB and (D) AVC groups; white arrows mark the EDD and ESD. KXB, ketamine-xylazine-treated burned rats; KXC, ketamine-xylazine-treated controls; AVB, avertin-treated burned rats; AVC, avertin-treated controls; EDD, end diastolic diameter; ESD, end systolic diameter.

**Figure 3:**
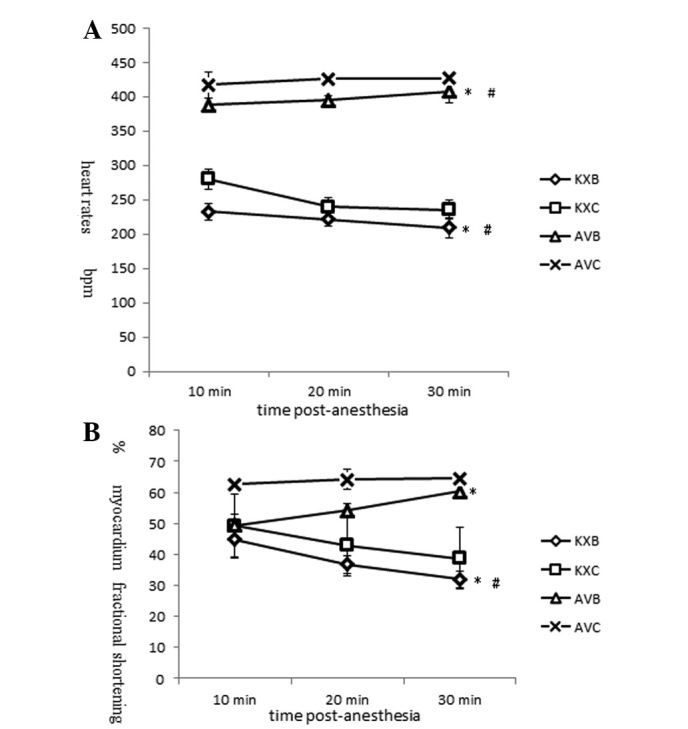
Echocardiographic measurements of heart rates and fractional shortening. KXB, ketamine/xylazine-treated burned rats (n=10); KXC, ketamine/xylazine-treated controls (n=10); AVB, avertin-treated burned rats (n=10); AVC, avertin-treated controls (n=10). ^*^Compared with the sham scald group (P<0.05), ^#^compared with the avertin group (P<0.05).

**Figure 4:**
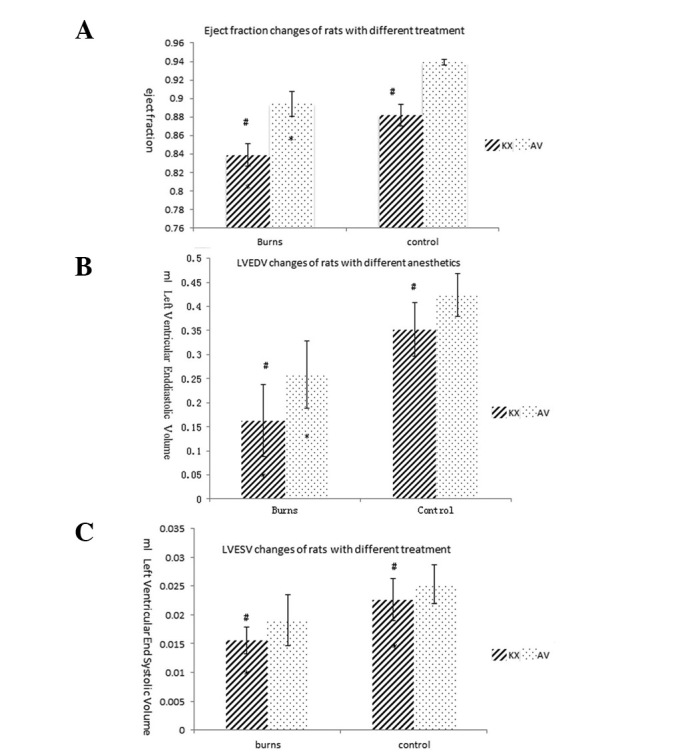
Echocardiographic measurements of ejection fraction (EF), left ventricular end-diastolic volume (LVEDV) and left ventricular end-systolic volume (LVESV). Changes of (A) EF, (B) (LVEDV) and (C) LVESV. ^*^Compared with the sham scald group (P<0.05), ^#^compared with the avertin group (P<0.05); KX, ketamine/xylazine; AV, avertin.

Ultrasonic cardiograms of the rats are shown in Fig. 2. The effects and time trends of K/X and AV on heart rates are shown in Fig. 3A. Rats anesthetized with K/X showed a significant reduction in HR (233±18.4 to 210±8.7 beats/min, P<0.05) over the 30-min period. The HR at each time point was significantly lower than that in AV group (P<0.01). The HR in the AV group was observed to increase slightly from 418±12 to 427±15 beats/min. The cardiac contractility results showed that FS% was inhibited and dropped acutely with K/X anesthesia (49.3±3.6 to 38.8±2.4%, P<0.01), but it remained stable with AV anesthesia (62.6±0.9 to 64.5±1.8%, P<0.05; Fig. 3B). Similar trends were observed for the EF, LVEDV and left ventricular end-systolic volume (LVESV; Fig. 4).

### Comparison between the two anesthetic regimes in severely burned rats

The HRs in the burned groups were significantly lower than those in the sham groups treated with K/X or AV (P<0.01). The HRs in the KXB group were fatally low, appeared to decrease during the study period and were significantly lower than those in the sham injury group. The FS% of the scalded rats in the KXB and AVB groups decreased to a significantly lower level than that in the AVC group rapidly within ten minutes after scalding (P<0.01). During the anesthesia period, FS% showed opposite time trends in the burned groups treated with K/X and AV. The FS% in the KXB group decreased, whereas that in the AV group was restored rapidly (Fig. 4). The EF in the burned group anesthetized with K/X was extremely low initially and remained low; the level was lower than that in the AVC and AVB groups (0.8387±0.03032 vs. 0.8942±0.03279 and 0.9392±0.00665, respectively). The scalded groups exhibited a smaller LVEDV and LVESV, as estimated by the Teichholz formula, than the sham groups (P<0.01). The LVESV (0.0156±0.0023 cm^3^) in the KXB group reached lower levels compared with those in the other three groups (KXC, 0.0226±0.0036; AVB, 0.0191±0.0044; and AVC, 0.0253±0.0033 cm^3^, P<0.05). The LVEDV in the KXB group (0.163±0.0743 cm^3^) was significantly reduced compared with those in the KXC, AVB and AVC groups (0.3517±0.05562, 0.2586±0.07048 and 0.4217±0.04411 cm^3^, respectively).

### Level of cTnI

**Figure 5:**
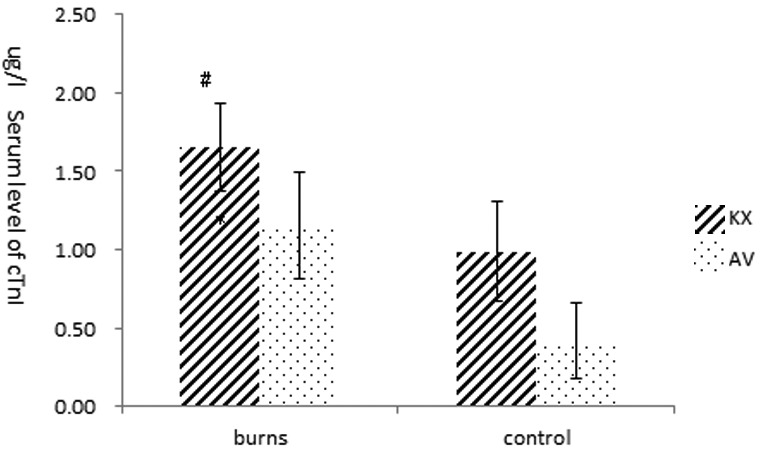
Serum cTnI levels in the rats. ^*^Compared with the sham scald group (P<0.05), ^#^compared with the avertin group (P<0.05). KX, ketamine/xylazine; AV, avertin.

Significant differences in serum cTnI levels were observed among the groups (P<0.01). The cTnI level in the KXB group (1.66±0.28 ng/ml) were significantly increased and was higher than the level in the other groups (KBC, 0.99±0.32; AVB, 1.16±0.34; AVC, 0.42±0.24 ng/ml, P<0.01). The cTnI level in the AVB group was slightly higher than that in the AVC group (P>0.05). No significant difference in cTnI levels was observed between the KXC and AVB groups (P>0.05; Fig. 5).

### Lung wet/dry weight and heart histopathological examination

**Figure 6:**
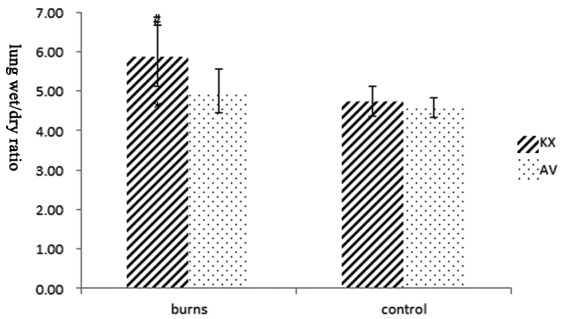
Lung wet/dry weight ratio (W/D) in rats. ^*^Compared with the sham scald group (P<0.05), ^#^compared with the avertin group (P<0.05). KX, ketamine/xylazine; AV, avertin.

To assay the index of pulmonary congestion of each group, the W/D was calculated. The lung W/D (Fig. 6) increased in the burned groups. The W/D in the KXB group (5.88±0.77) increased significantly and was higher than those in the KXC, AVB and AVC groups (4.74±0.37, 5.00±0.55 and 4.59±0.26, respectively, P<0.01). No significant difference in W/D was observed between the KXC and AVC groups (P>0.05).

**Figure 7:**
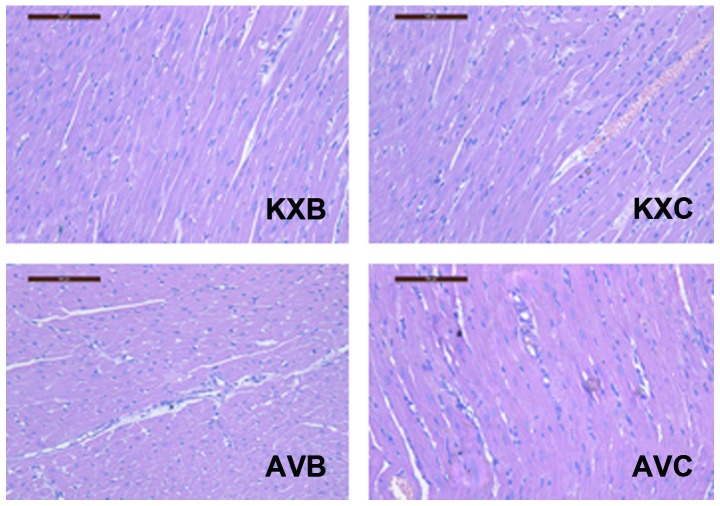
Histopathology of heart tissue. KXB, scalds anesthetized with ketamine/xylazine; KXC, sham scalds anesthetized with ketamine/xylazine; AVB, scalds anesthetized with avertin; AVC, sham scalds anesthetized with avertin. Hematoxylin & eosin staining, magnification, ×100.

The histopathology of heart tissue from each group is shown in Fig. 7. No degeneration of the myocardium was observed in any group. A certain amount of inflammatory cell infiltration was observed; however, this is common in rodents.

### Apoptotic cell death by triggering the mitochondrial pathway

**Figure 8:**
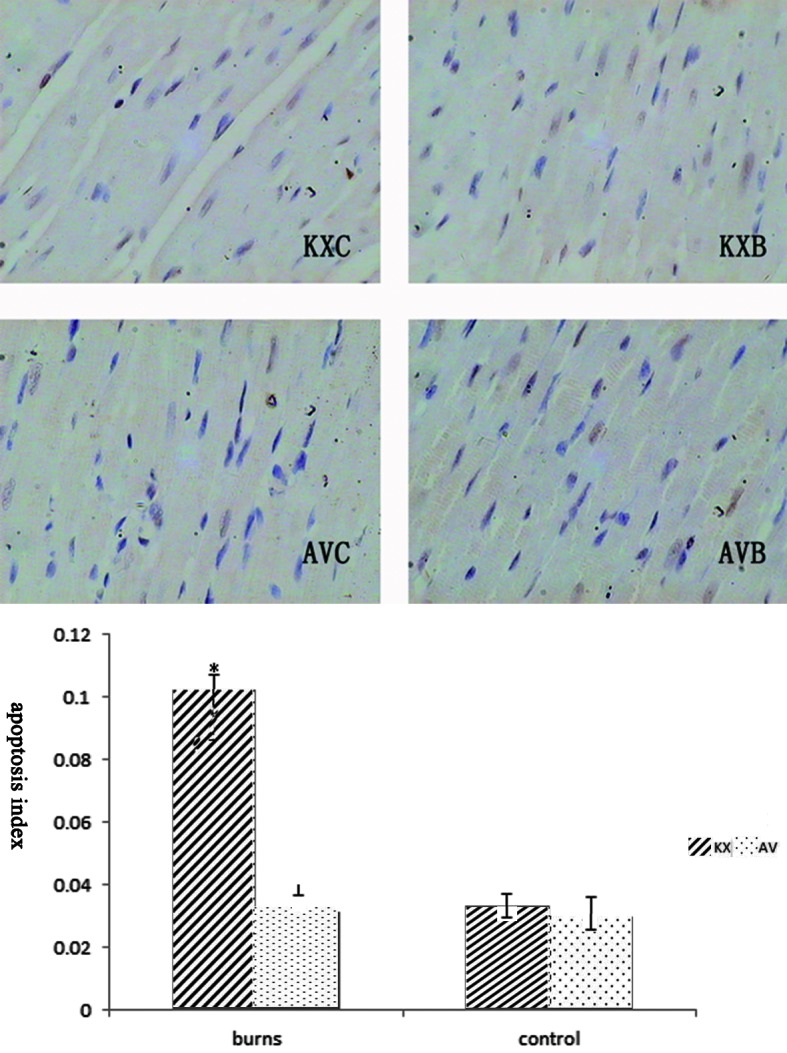
Apoptotic nuclei determined by TUNEL. ^*^Compared with the sham scald group (P<0.05). KXB, scalds anesthetized with ketamine/xylazine; KXC, sham scalds anesthetized with ketamine/xylazine; AVB, scalds anesthetized with avertin; AVC, sham scalds anesthetized with avertin; KX, ketamine/xylazine; AV, avertin. Hematoxylin & eosin staining, magnification, ×400.

**Figure 9:**
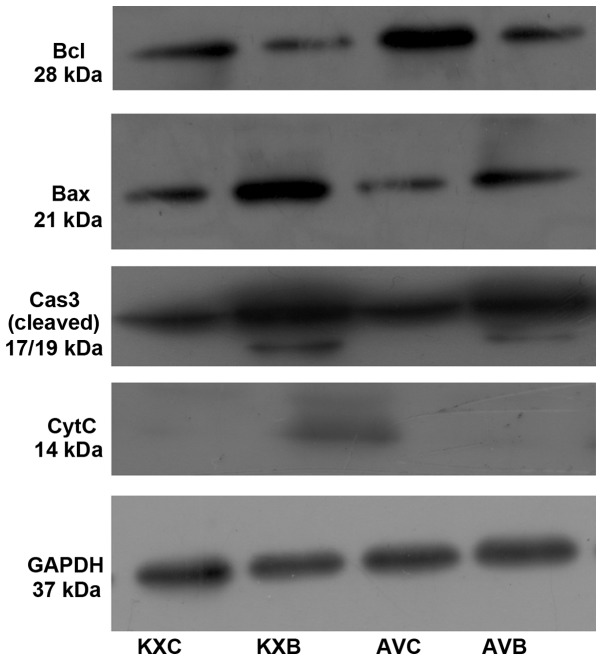
Apoptosis-related protein levels determined by western blotting. KXB, scalds anesthetized with ketamine/xylazine; KXC, sham scalds anesthetized with ketamine/xylazine; AVB, scalds anesthetized with avertin; AVC, sham scalds anesthetized with avertin; cas3, caspase 3; CytC, cytochrome *c*.

**Figure 10:**
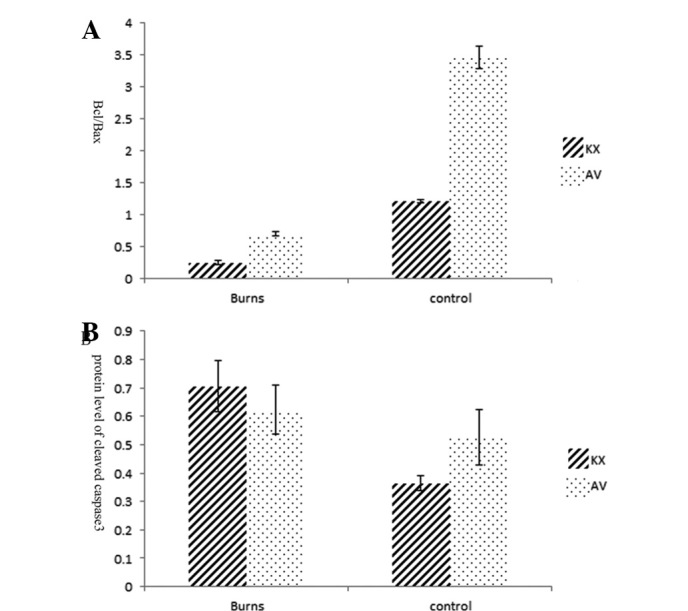
Expression of proteins of the mitochondrial apoptosis pathway. (A) Bcl/bax ratio changes and (B) cleaved caspase concentration changes in rat hearts. KX, ketamine/xylazine; AV, avertin.

To analyze the development of apoptosis following scald injury, heart tissue was analyzed by the TUNEL technique (Fig. 8). As the figure shows, apoptotic nuclei were more evident with irregular contours in the cardiac myocytes of scalded rats, although there was no significant difference between the AVB and AVC groups (P>0.05). The expression of mitochondrial apoptosis-related proteins is shown in Fig. 9. There was a 3-fold increase in the apoptotic index of myocardial cells in the KXB group compared that in the AVB group (P<0.01). The activation of the mitochondrial apoptosis pathway is indicated by the expression of cytochrome c and the ratio of bcl/bax, the latter was much lower in group KXB than in the other groups (P<0.01; Fig. 10A). The level of executioner caspase 3 (cleaved caspase 3), increased in the scalded rats. There was a more than 2-fold increase in the caspase 3 concentration in the KXB group compared with that in the KXC group (P<0.01), and a more than eightfold increase in the AVC group than that in the AVB group (P<0.01; Fig. 10B).

## Discussion

Cardiac dysfunction is one of the most common complications of severe burns. Anesthetics, however, have an impact on cardiac function. In the present study, the combined impact of burn injury and anesthetics was explored. Echocardiographic measurements and blood biochemical examinations were used to show that anesthesia may have significant effects on cardiac function, which are more harmful in individuals with severe burns. The mitochondrial apoptosis pathway may be involved in this process. These findings have important implications for the selection of an appropriate anesthetic during study of severe burns in rats.

Ketamine, which is a phencyclidine derivative, is commercially available in a racemic form or as an S(+) purified isomer. The racemic form consists of a mixture of S(+) and R(−) isomers. Ketamine activates the sympathetic system, resulting in an increase in HR, cardiac output and oxygen consumption. Initially, ketamine is distributed to highly perfused tissues. Due to these significant side-effects, ketamine may be combined with tranquilizers or sedatives, such as xylazine, to provide a relatively safe anesthesia that may be administered without specialized equipment. Xylazine counterbalances the undesirable effects of ketamine, however, its use may cause cardiovascular abnormalities arising from a reduction in sympathetic tonus (23).

In the current study, we observed that the K/X combination markedly suppresses cardiac function, characterized by reductions in HR, FS% and LVEDV, even in normal rats. These results are consistent with those of a previous study which identified that K/X anesthesia resulted in the most prominent negative inotropic and chronotropic responses compared with pentobarbital and isoflurane anesthesia (24). Our data showed that K/X had a greater HR inhibitory effect than that reported in the previous study (240±7 vs. 326±4 beats/min). AV showed slight effects on the HR, which was similar to that observed for the unanesthetized rats (427±10 vs. 421±26 beats/min). In addition, other parameters, including FS%, EF and LVEDV, also changed only slightly, which indicated that AV produced only a slight cardiac inhibitory effect. This result was validated by the W/D and the cTnI level.

Reduced cardiac function as a major component of multi-organ failure following burn injury was recognized as early as 1931 (25). In the present study, the contractility and diastolic functions, including FS%, EF, LVESV and LVEDV, also indicated there was a depressed cardiac state in scalded rats. No definite signal pathway has been confirmed to be responsible for cardiac dysfunction following injury. Two major theories have been proposed for the pathogenesis of myocardial dysfunction following burns. One involves decreased myocardial perfusion due to hypovolemia following burns, which leads to ischemic injury and results in direct cardiomyocyte damage. The second theory involves the inflammatory or toxic response, which mainly induces reversible intrinsic myocardial depression, by cytokines or lipopolysaccharides (26,27). In the adult mammalian heart, cardiac myocyte apoptosis has been identified as a mechanism of cell death in acute myocardial infarction and ischemia-reperfusion injury. Lightfoot *et al*(17) reported that there was a 10-fold increase in the number of apoptotic cells in the subendomyocardium of the left ventricular tissue harvested 24 h post burn compared with the number of apoptotic cells in sham burn controls. We observed that the number of apoptotic nuclei increased and was accompanied by activation of the mitochondrial pathway. However, it is not known whether the occurrence of apoptosis actually contributes to the development of the heart dysfunction.

In preliminary experiments, we observed that rats anesthetized with K/X (25/6 mg/kg) had high mortality following severe burns, such as those covering 30% TBSA. Pleural effusion was observed in the rats that died within 24 h. Therefore, we designed the current study to explore the cardiac impacts of anesthesia and burns. We also used echocardiography and the cTnI assay to identify abnormal cardiac parameters that have been used for the diagnosis of myocardial damage in other models (28). Echocardiography was used to show that the burned rats had lower HRs, FS%, EF and LVEDV than the unburned rats. Several authors have described the association between cardiac dysfunction and severe burns during the shock stage in various models (29,30).

We observed that cardiac function in the burned rats anesthetized with K/X was inhibited more severely compared with that in the burned rats anesthetized with AV. The same procedure of scalding and resuscitation was applied in the KXB and AVB groups. Therefore, the difference between these two groups likely resulted from the anesthetics, ketamine and xylazine, which produced negative impacts on these rats. Under normal conditions, ketamine stimulates the sympathetic system by increasing circulating catecholamine concentrations (31). Ketamine, however, exhibits potentially negative cardiovascular effects in patients with catecholamine-dependent heart failure (32) or other critical illnesses in which catecholamine is excessively mobilized. Therefore, it may be that the stress of severe burns weakened the sympathetic stimulation and therefore, the cardiac inhibition effect of ketamine became dominant in severe thermal injury.

It has been clinically confirmed that there is a close correlation between echocardiography and leakage of troponin (33). Cardiac troponin T and cTnI are now recognized as the most tissue-specific and sensitive biomarkers associated with cardiac damage and have been included as a diagnostic criterion for several cardiac-related pathologies (34,35). A high level of troponin may result from increased permeability of the myocytes or degradation of native troponin into smaller fragments due to ischemia (36). Franco *et al* reported that changes in creatine kinase isoenzyme fraction MB (CK-MB) serum activity observed in dogs treated with atropine, xylazine and ketamine S(+) were higher than the baseline values 6 h after the experiment (37). These data indicate that there is likely to be a definite change in cTnI levels. The cTnI level 24 h after burns has been reported to be significantly higher in patients with burns covering >30% TBSA and cTnI has been regarded as a marker for post-burn cardiac injury (38). Therefore, we measured the changes in cTnI levels to assess the impact of anesthetics on burn injury. It was observed that the cTnI levels increased in the burned groups and were particularly high in the rats anesthetized with K/X, a result that indicated greater heart injury. A possible explanation for these findings is that K/X depressed cardiac function and reduced cardiac output, which exacerbated the hypoperfusion of coronary circulation caused by severe burns. Thus, ischemia and oxygen deficiency may cause myocardial damage.

Pulmonary edema is one of the most significant syndromes of acute heart failure. The simplest way to evaluate edema formation in the lung is to use gravimetric approaches, such as W/D. A much higher W/D was observed in the KXB group.

As previously mentioned, burns triggered significant apoptosis in the myocardium, which indicated that the apoptosis signaling pathway and the caspase family proteases may be significantly involved in the development of myocardial dysfunction following thermal injury. In the present study, results indicative of apoptosis were observed and suggest that mitochondrial apoptosis may be involved as the parameters of apoptosis, such as the bcl/bax ratio and cytochrome *c* level, changed significantly; these effects were worsened by anesthetics such as the K/X combination.

At first, we hypothesized that differences in the experimental systems may have caused this outcome; for example, the proportion of xylazine in the K/X combination in the present study (25/6 mg/kg) was higher than those in other studies [37/7 mg/kg (23), 80/10 mg/kg (39) and 100/4 mg/kg (40)], which may be an additional cardiac inhibitory factor. The K/X combination, however, has been used widely and safely, although K/X has been reported to be associated with more highly elevated levels of cytokines, such as IL-6, than are associated with isoflurane in rats with burn injury (39). In a recent study, a high dose of K/X (200/60 mg/kg) was even observed to significantly reduce myocardial infarct size compared with the low dose, and may introduce unwanted variability in ischemia-reperfusion studies (41). Therefore, the evident inhibitory result of K/X in this study is considered to be mainly due to the synergistic effect of anesthetic and burn injury.

Although certain serious side-effects have been reported, the safety and efficacy of AV in mice are well-documented. AV caused only a modest reduction in LV FS%, and was similar to that of the conscious rats.

In summary, we recommend that particular attention be given to the choice of anesthetic drugs used in experiments studying burn injury. Although it remains difficult to recommend an optimal choice of drugs and anesthesia technique for different models and animal species, our results suggest that the K/X mixture causes evident cardiac inhibition in severely scalded rats. AV may be the optimal choice for animal models with severe burns.
